# Nanoindentation of Soft Biological Materials

**DOI:** 10.3390/mi9120654

**Published:** 2018-12-11

**Authors:** Long Qian, Hongwei Zhao

**Affiliations:** School of Mechanical and Aerospace Engineering, Jilin University, Changchun 130025, China; qianlong17@mails.jlu.edu.cn

**Keywords:** nanoindentation, mechanical properties, soft biomaterials, viscoelasticity, atomic force microscopy (AFM)

## Abstract

Nanoindentation techniques, with high spatial resolution and force sensitivity, have recently been moved into the center of the spotlight for measuring the mechanical properties of biomaterials, especially bridging the scales from the molecular via the cellular and tissue all the way to the organ level, whereas characterizing soft biomaterials, especially down to biomolecules, is fraught with more pitfalls compared with the hard biomaterials. In this review we detail the constitutive behavior of soft biomaterials under nanoindentation (including AFM) and present the characteristics of experimental aspects in detail, such as the adaption of instrumentation and indentation response of soft biomaterials. We further show some applications, and discuss the challenges and perspectives related to nanoindentation of soft biomaterials, a technique that can pinpoint the mechanical properties of soft biomaterials for the scale-span is far-reaching for understanding biomechanics and mechanobiology.

## 1. Introduction

Mechanical behavior of biological materials has come to the front stage recently, not only since its importance from the mechanical and load-bearing viewpoints, but also in the way that it influences other bio-functionalities [1]. Recent studies have directly linked major biological performances, mechanisms, and diseases to the mechanical response from the biomolecular up to the organ level [2,3,4,5,6,7]. In addition to the many medical applications, mechanical characterization of biological materials has also fueled the recent growth of materials science and engineering applications—bionics [8], whereas the mechanical characterization of soft biomaterials, especially down to biomolecules, is more difficult and fraught with more pitfalls compared with the hard biomaterials.

At present, aiming at characterizing soft biomaterials, a variety of testing techniques have been developed and utilized widespread from bulk scale to the micro/nano-scale [9,10,11,12,13,14]. While every technique has its pros and cons, nanoindentation (including AFM) is considered as a powerful tool to conduct mechanical analyses, especially down to micro/nano-scale with nanometer depth and sub-nanonewton force resolution [15]. Firstly, and the most attractively, its micro/nano-mechanical contact methodology allow for region-specific mapping of biomaterials inhomogeneity [16,17], and studying of cell mechanics [18]. Secondly, its unrestriction of tissue morphology avoids special preparation of material samples, which can be taken advantage of in the field of in vivo testing [19,20]. Thirdly, its capacity of coupling with other optical techniques provides a new horizon for mechanical characterization [21,22]. Fourthly, compared with traditional testing methods with a single-measurement mode, indentation can provide an ideal loading modality whose stress and strain fields comprise tension, compression, and shear loading modes [23]. Further, as a non/micro-invasive method, nanoindentation can also be utilized for some valuable samples, such as fossils that are millions of years old [24].

In regard to nanoindentation of soft biological materials, current measurements lack a standardized testing routine owing to several challenges we are facing:

1. Soft biomaterials are significantly less stiff than typical engineering materials, which may be to some extent in conflict with the range/resolution of some commercial instruments.
2. Most nanoindentation instrumentations are designed based on “dry state” testing, lacking environmentally-controlled components to build physiological environment (such as fluid submersion and thermal control).
3. For most commercial instruments with the optical microscopes, the interference between the indenter/microscope and the sample surface may exist in the switch (movement) of indenter/microscope owing to non-ideal surface. This may lead to a considerable challenge, particularly in in vitro and in vivo testing.
4. There is a paucity of consensus on the appropriate data analysis to mechanical characterization of soft biomaterials.

In addition to the above factors, some features of soft biomaterials, such as viscoelasticity and adhesion, may also make some deviations when testing. These challenges have limited the development and utilization of nanoindentation technique which, in turn, slow the footsteps of characterization of soft biomaterials. In this review, we will discuss the above factors, and conclude with the perspectives and opportunities of nanoindentation of soft biomaterials.

## 2. Soft Biomaterials

**Figure 1:**
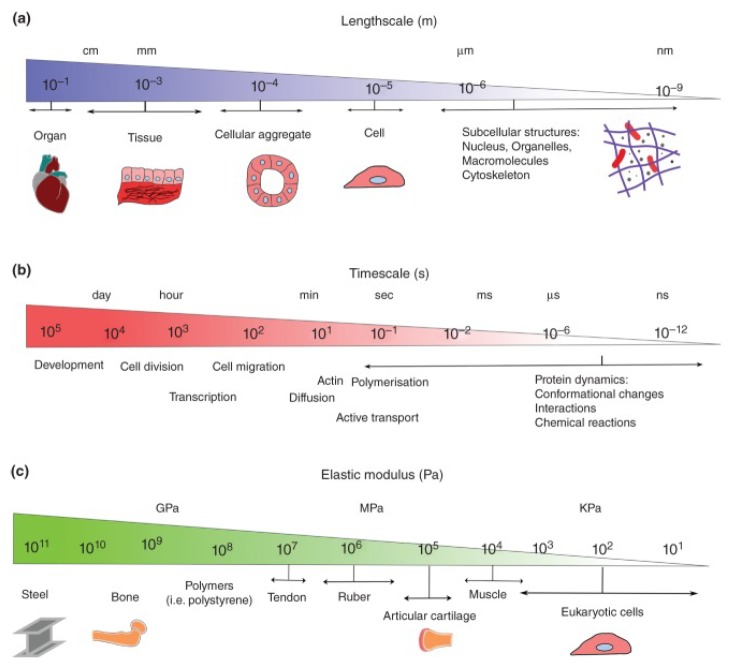
Multiple scales of soft biomaterials. (**a**) Length scales from the molecular to the organ level; (**b**) timescales of different physiological processes; and (**c**) comparisons of the elastic modulus among different typical materials. Reproduced with permission from [32].

For most people, soft materials are materials where the deformation can be felt by hand or seen with the naked eye without applying an excessive force [25], which reflects the feature in a straightforward manner: it is much more compliant than many engineering materials. In fact, the words “soft” and “hard” do not indicate anything in regard to hardness or plastic deformation exactly, and soft biomaterials only imply non-mineralised in their healthy state [26]. Soft biomaterials, such as globular proteins [27], cancer cells [28], arteries [29], cartilage [30], and the brain [31], vary in multiple length scales from the molecular via the cellular and tissue all the way to the organ level, and the above complex hierarchical structures make characterization within wide physiologically-relevant timescales and the elastic modulus range (Figure 1).

A number of constitutive models, such as linear elastic, hyperelastic, viscoelastic, and poroelastic models, have been widely used to mechanical characterization of soft biomaterials. In fact, these models can fall into different aspects of the constitutive response, in particular that distinguish them from typical engineering materials.

**Figure 2:**
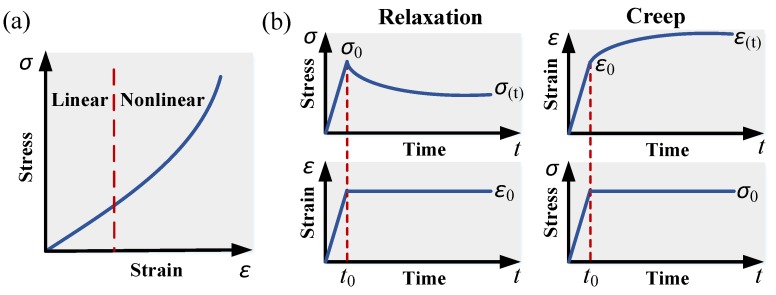
Constitutive responses of soft biomaterials. (**a**) Nonlinear stress–strain (*σ* − *ε*) behavior, in which the stress profile can be considered linear under small strain; and (**b**) time-dependent mechanical behavior: stress-relaxation (**left**) and creep (**right**).

First, many soft biomaterials exhibit nonlinear stress–strain (*σ* − *ε*) behavior, with an increasing stiffness as the strain increases. Nonetheless, the stress can be considered proportional to the strain under small deformations (Figure 2a), in which the relationship between stress and strain is governed by a constant elastic modulus *E* (just as the elastic stage of typical engineering materials), and can be fitted to linear elastic model (*σ* = *Eε*)—the simplest type of material response. The threshold of the linear range varies from different biomaterials and testing methods [33], and further discussion of the linear level can be seen in Section 4.3. Another strategy of simplification of nonlinear behavior with the linear elastic model is to divide the stress profile into several portions. At each portion, the mechanical behavior can be considered linear and characterized with the linear elastic model [34].

To acquire more accurate constitutive data, hyperelasticity provides a means of modeling the nonlinear stress–strain behavior of soft biomaterials. Many different kinds of hyperelastic models could be used to characterize mechanical behavior, such as Neo–Hookean, Mooney–Rivlin, Ogden, and Yeoh models [35,36]. As far as the above models are concerned, the Ogden model is classically used for finite element simulations, and Neo–Hookean, Mooney–Rivlin, and Yeoh models are best used at low, moderate, and high strain levels, respectively [37]. More details about different hyperelastic models and their parameters determined from indentation can be found in the literature [37,38].

The second aspect of soft biomaterials is the time-dependent mechanical behavior, i.e., viscosity. Viscoelastic model, displaying a combination of both elastic and time-dependent responses. A viscoelastic material stores and dissipates mechanical energy simultaneously undergoing imposed mechanical excitation, with the response of stress-relaxation or creep over time, as shown in Figure 2b. Typically, the model formulated by a Prony series approximation can be used to describe viscoelastic response:

(1) $$G(t)=G_{\infty }+\sum_{j}G_{j}\cdot e^{-t/\tau_{j}}$$

where *G_∞_* is the equilibrium shear modulus, *τ_j_* is the time constant for each exponential term, and *G_j_* is the associated magnitude of shear modulus. The initial shear modulus can be calculated by summing *G_∞_* and *G_j_*. The above parameters can be predicted using a minimization algorithm [39]. Additionally, nonlinear viscoelasticity can also been modeled during indentation [40].

As for most soft biomaterials, they are also hydrated, and a poroelastic (also called biphasic) model can also be used to describe time-dependent behavior under physiological condition: the flow of a fluid through a porous elastic solid [41]. In addition to the shear modulus *G* and the (drained) Poisson’s ratio *υ* used to characterize the elastic behavior of the porous skeleton, Darcy (hydraulic) permeability *κ*, formulated between the fluid viscosity *η* and the intrinsic permeability *k* (*k* = *κη*), is an important parameter to indicate the fluid–solid coupling and the fluid flow [42]. More details about poroelasticity during nanoindentation of soft biomaterials will be discussed in the Section 4.5. 

Another aspect of soft biomaterials is the anisotropy and the heterogeneity. Some soft biomaterials exhibit direction-dependent and region-specific behavior based on their structural complexities at multiple length scales [17,43,44]. Apart from inherent complexities of materials, some external conditions may also lead to the above behavior [45,46]. It is imperative to characterize this structure-mechanical behavior with some specific analysis methods and models [46,47,48].

## 3. Methods

Two kinds of commercial instruments have been developed and utilized widespread for indentation testing, especially down to micro/nano-scale: dedicated instrumented indentation instruments (nanoindenter) and the atomic force microscope (AFM). In this section, we will introduce the working principles of two different instruments, and discuss the analysis of experimental data based on the force-indentation curves.

### 3.1. Nanoindenter

**Figure 3:**
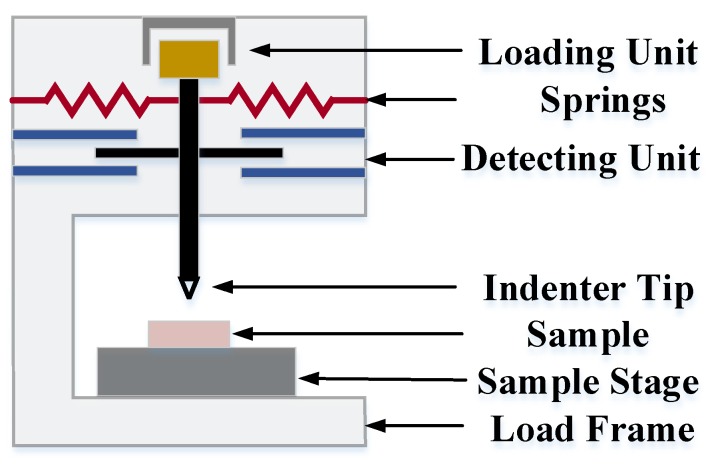
Schematic diagram of a nanoindenter instrument.

Nanoindentation, a form of depth-sensing indentation (DSI) testing technique, involves the application of a controlled load/depth to the surface to induce local surface deformation (Figure 3). During a typical testing, Load *P*, indentation depth *h* and time *t* are monitored as the indenter is actuated into the test material’s surface. The response of *P* − *h* − *t* trace is fitted to a range of different constitutive models to identify mechanical properties of the sample.

Usually, a nanoindenter consists of several essential components:

- Loading unit: typically actuated by the expansion of the piezoelectric element, magnetic coils, or electrostatically [49].
- Detecting unit: sensors (capacitance or inductance) to record the displacement of the indenter. In fact, whether the strategy applying force and measuring displacement through separate means, or using the same transducer, the data of raw force and displacement are always coupled due to the leaf springs [50].
- Indenter tip: for soft biomaterials, typically using dull indenters (such as spherical and flat-ended), rather than sharp indenters (such as Berkovich and Vickers) to avoid penetration of the sample.
- Sample stage: a two- or three-coordinate stage (*x*, *y*, or *z*) to move the sample.
- Microscopes: to observe and choose the point of the sample during testing.
- Control system: the computer with the software to operate instrument, analyze results, and save data.
- Other additive components: such as custom irrigation system [51] and fluid cell [52] for biomaterials.

Definitely, the design of instruments may be not identical to the above components, especially for the custom instruments, but part of them and similar working principles are, at least, included [31,53].

### 3.2. Atomic Force Microscopy (AFM)

Atomic force microscopy (AFM), a part of the scanning probe microscopy branch, operates based on the interaction between the sample surface and the small tip located on the end of a sensitive cantilever. Apart from the basic goal of imaging surface morphology, AFM can also act as a powerful instrument to conduct micro/nano-mechanical analysis. In particular, the mode of mechanical characterization combined with high resolution imaging technique, allows a more targeted investigation of biomaterials features, especially down to nanoscale.

**Figure 4:**
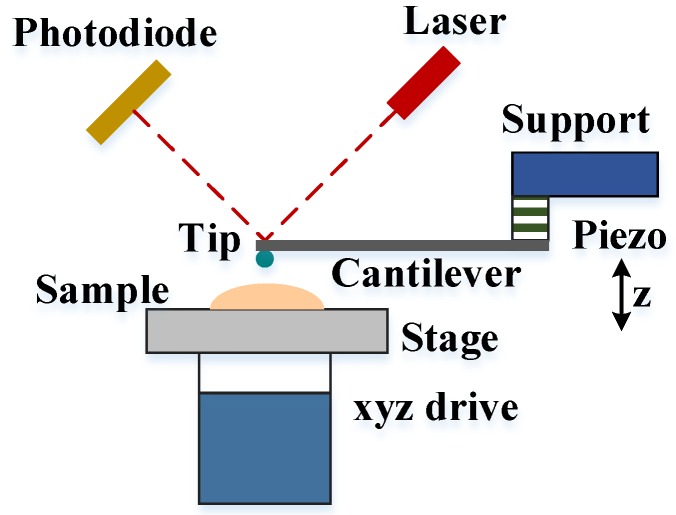
Schematic diagram of an AFM instrument.

The setup and the measurement of AFM have been covered in a number of works [54,55,56], and a brief introduction is given here. During typical testing, the tip or the sample is moved to each other with a piezoelectric, until the tip-sample contact occurs and the cantilever deflects (Figure 4). The indentation force *P* can be described by Hooke’s law:

(2) $$P=k_{c}\Delta d$$

where *k_c_* denotes the spring constant of the cantilever, and ∆*d* is the corresponding deflection of the cantilever.

For stiff samples, which are several orders of magnitude stiffer than the tip, the displacement of the piezo ∆*z* is equal to corresponding deflection of the cantilever ∆*d* (∆*z* = ∆*d*), since the indentation depth *h* is zero (*h* = 0). However, for many samples (e.g., soft biomaterials), the displacement of the piezo ∆*z* is larger than the corresponding deflection of the cantilever ∆*d* owing to the indentation, and the indentation depth *h* can be expressed as following:

(3) h=Δz−Δd

So far, the “deflection–displacement curve” of AFM indentation can be developed to typical “force-indentation (*P* − *h*) curve” in nanoindentation, and analyzed likewise by different constitutive models.

### 3.3. Force–Indentation (P − h) Curves

#### 3.3.1. Oliver–Pharr Model

The analysis of force–indentation (*P* − *h*) curves of commercial nanoindnetation system is often based on the work by Doerner and Nix [57] and Oliver and Pharr [58]. It is assumed that only the elastic recovery occurs in the initial portion of the unloading, while the loading is an elastic–plastic contact mode. The relationship formulated between indentation force *P* and depth *h* during unloading can be expressed a power law relation:

(4) $$P=\alpha {\left(h-h_{f}\right)}^{m}$$

where *h_f_* is the final residual indent of depth, *α* and *m* are power law fitting constants related to the indenter geometry. The stiffness *S*, defined as the resistance in response to an applied force, can be calculated by the slope of the upper portion of the unloading curve:

(5) $$S=\frac{dP}{dh}$$

Further, the reduced elastic modulus *E_r_* (the combined modulus of the tip and the sample) can be determined in the terms of the unloading the contact area *A*:

(6) $$E_{r}=\frac{\sqrt{\pi }}{2}\frac{S}{\sqrt{A}}$$

and the sample elastic modulus *E_s_* can be determined by decoupling the deformations of both indenter and the sample as given by:

(7) $$\frac{1}{E_{r}}=\frac{\left(1-v_{s}{}^{2}\right)}{E_{s}}+\frac{\left(1-v_{i}{}^{2}\right)}{E_{i}}$$

where *v* is the Poisson’s ratio, subscript *s* and *i* refer to the sample and indenter material respectively.

The above approach works very well for typical engineering materials and some hard biomaterials (e.g., bone [59]), but for the materials with time-independent mechanical responses (i.e., soft biomaterials here), Oliver–Pharr method is invalidated due to the creep to overwhelm the elastic recovery [60], resulting in a near-vertical or even negative slope in the initial unloading region. For this reason, some “corrections” have been adopted based on Oliver–Pharr analysis, such as high unloading rates [61], long hold periods [62] and the data analysis based on the measured creep rate [63,64,65]. Another limitation of Oliver–Pharr method is that the results necessarily rely on the contact area (tip area function), which may lead to significant errors in the cases of the “tip radius“ effect [66,67] and the pile-up (or sink-in) effect [68]. Accordingly, some correction factors [69,70] or new approaches [71] may be also taken into account to evaluate the actual properties of the material.

#### 3.3.2. Hertz Contact Model

Hertz model, the most well-known and applied theory in mechanical characterization of materials, was proposed by Hertz to solve the problem of contact between two smooth, ellipsoidal bodies [72]. Some assumptions employed for validity are required:

- Small contact area and small deformations.
- Isotropic and homogenous materials.
- Adhesionless and frictionless surfaces.

Following the above assumptions, Sneddon made a significant contribution to the theoretical framework to formulate the relationship between force and depth for a punch of arbitrary profile penetrated [73]. Initially, Sneddon’s solution was developed for elastic contact of hard materials, and it has been proved applicable to extend to determine the initial shear modulus and even hyperelastic parameters of non-linear soft materials [74].

**Figure 5:**
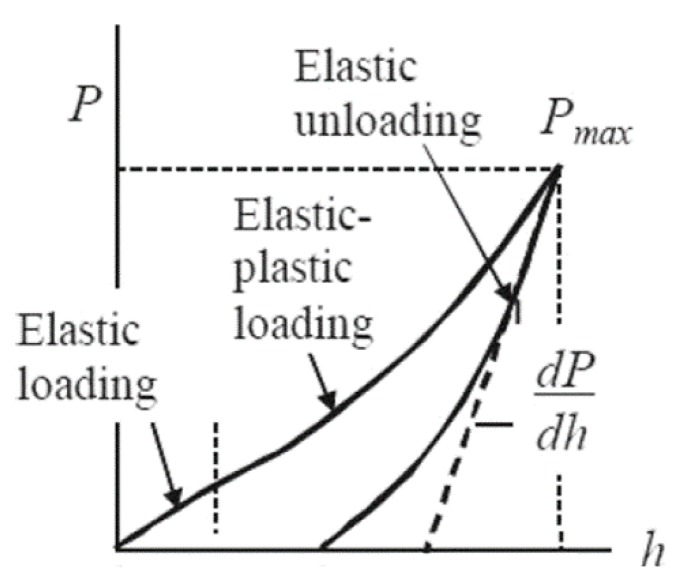
Typical force–indentation (*P* − *h*) curve for spherical indenter, in which the Oliver–Pharr model can be used in elastic unloading portion, and Hertz contact model can be used in elastic loading portion. Reproduced and adapted with permission from [49].

For the indentation by a flat-ended cylindrical indenter, the indentation force *P* and depth *h* can be directly related through the following equation:

(8) $$P_{\mathrm{cylinder}}=\frac{4R}{1-\nu }Gh$$

where *R* is the cylinder radius, and *G* and *υ* are the shear modulus and Poisson’s ratio of the soft biomaterial, respectively. For a spherical indenter (Figure 5), the indentation force is:

(9) $$P_{\mathrm{sphere}}=\frac{8\sqrt{R}}{3(1-\nu )}Gh^{3/2}$$

where *R* is the sphere radius, and for a conical indenter:

(10) $$P_{\mathrm{cone}}=\frac{4\tan\alpha }{\pi (1-\nu )}Gh^{2}$$

where *α* is the cone half angle.

Sometimes, the above equations may need to be modified owing to the non-ideal effects while mechanical characterization [31,75], such as the correction factor of size effect [76,77] and the compensating factor for the tip [78,79]. When it comes to micro/nano-scale (such as cell indentation), the above effect may be much more significant considering that the indentation depth is comparable to the cell dimension. The Hertz contact model may become invalid owing to large deformation or thin-layer effect, in which case some correction factors or new models need to be taken into account [80,81,82].

For the characterization of time-dependent behavior, a Boltzmann hereditary integral, based on associated solution for a linearly elastic material, was proposed to capture the time-dependent stresses and deformations along the total temporal scale [83]. For the flat-ended cylindrical indenter with a radius *R*, applying the hereditary integral in Equation (8), the time-dependent indentation force *P*(*t*) and depth *h* relation is represented by:

(11) $$P_{\mathrm{cylinder}}(t)=\frac{4R}{1-\nu }\int_{0}^{t}G(t-\tau )\;(\frac{dh}{d\tau })\;d\tau$$

Similarly, for the spherical indenter with a radius *R*:

(12) $$P_{\mathrm{sphere}}(t)=\frac{8\sqrt{R}}{3(1-\nu )}\int_{0}^{t}G(t-\tau )\;(\frac{dh^{3/2}}{d\tau })\;d\tau$$

and for the conical indenter with a cone half angle *α*:

(13) $$P_{\mathrm{cone}}(t)=\frac{4\tan\alpha }{\pi (1-\nu )}\int_{0}^{t}G(t-\tau )\;(\frac{dh^{2}}{d\tau })\;d\tau$$

In Equations (11)–(13), the time-dependent shear modulus *G*(*t*) can be expressed by a linear viscoelastic model (see Equations (1)) to characterize time-dependent mechanical properties [84,85].

Overall, compared with the Oliver–Pharr model based on elastic–plastic deformation (or the “correction” based on viscous-elastic–plastic deformation), the Hertz and Sneddon models, based on elastic contact, may dominate the literature for soft biomaterials due to their simplicity and widespread application [86]. The comparisons between the Oliver–Pharr method and Hertz contact model are summarized in Table 1, in which some aspects, such as tip selection and control mode, will be further discussed in the next section. The reader is also encouraged to review more analytical approaches in/beyond the Hertzian regime provided in the literature [87].

## 4. Test Protocols

Compared with the typical engineering materials and hard biomaterials, soft biomaterials are much more compliant, with the elastic modulus values down to MPa or even kPa range. Considering the inherent complexities of soft biomaterials, great care and consideration are required to characterize mechanical behavior at multiple scales, especially when utilizing commercial instrumentation. In this section, some limitations as to data collection and analysis are considered, and a set of guidelines are presented.

### 4.1. Tip Selection

#### 4.1.1. Tip Material

As is well-known, the materials of indenter tip are relatively stiff compared with the sample, to ensure that the compliance of tip is tiny and can be ignored. Owing to the low modulus of soft biomaterials, tip materials can be changed from the diamond or sapphire, which is widely used in typical nanoindentaion, to steel [88], aluminum [33], or even silica [89] and silicon [31].

#### 4.1.2. Tip Geometry

For indenting soft biomaterials, whose force–indentation regime is opposite to stiff materials, small forces arise at relatively large displacements compared with large forces at relatively small displacements [90]. Thus, a dull tip (spherical [43] and flat-ended [33]) is commonly used instead of a sharp tip (Berkovich [91], Vickers [92], conical [93], and cube corner [94]). Some advantages of a dull tip are as follows:

- A dull tip can achieve a larger contact area and therefore a higher force level compared with a sharp indenter at the same indentation depth, which allows for the testing within range/resolution of the instrument.
- The high plastic deformations and stress concentrations induced by a sharp tip could lead to sample damage (from tissue penetration to cell membrane rupture) and excess of linear elastic limit.
- Considering heterogeneity and complexity of soft biomaterials inherently, a sharp tip may lead to significant differences among repetitive tests owing to too small tip-sample contact area. Thus, one measurement with a dull tip can be considered as the average of many measurements with the sharp probe, and consequently, less time to obtain robust statistics.

In spite of the wide application of spherical and flat-ended tips for characterizing soft biomaterials, each one has its own advantages and drawbacks, and may be more applicable to specific conditions, as shown in Table 2. The reader is also encouraged to review more details about different tips in the literature [82,95].

#### 4.1.3. Tip Dimension

By virtue of different tip dimensions, indentation instruments can provide the versatility of functioning across the length spectrum ranging from bulk scale to the micro/nano-scale, and even large-scale indentation (with millimeter-sized or larger tips) has also been widely used for characterization of some extremely soft biomaterials (e.g., brain tissue [33]). Anyway, to avoid complicating the interpretation of the data, a criterion is suggested here to be exercised while indentation: the size of tip *R* << current length scale level of the measured sample *L*_current level_, and tip size *R* >> the lower length scale level *L*_lower level_. For example, if the goal is to characterize the mechanical behavior at tissue-level, the tip size need to be much small compared with the tissue, to adapt to the Hertz assumption of infinite half space (*R* << *L*_tissue level_), as well as larger than the diameter of an individual cell or fiber (*R* >> *L*_cell level_).

### 4.2. Control Mode

For most commercial nanoindenters, a load-controlled mode is utilized by default. However, for soft biomaterials which are extremely compliant, adhesive and time-dependent, this mode can be difficulties with the contact detection and data analysis, in which case displacement-control is extremely useful.

Firstly, displacement-control can overcome unambiguous tip-sample contact detection. Whether the contact is ascertained by a small force change or a small apparent stiffness change in the mode of load-control, the characteristics of low modulus and time-dependence of soft biomaterials would play against the change of force and stiffness respectively. Accordingly, tests starting below the sample surface detection may happen, leading to significant overestimations of the elastic modulus of soft materials via indentation [96]. On the contrary, the test can be initiated prior to tip–sample contact in the displacement-controlled mode, when the probe is slightly above the sample, and some correction of the contact point can be applied post-hoc.

Additionally, displacement-control can simplify data analysis of time-dependent behavior. For most soft biomaterials, they are viscoelastic materials whose properties depend on the strain rate. In the mode of load-control, the strain rate is not a constant during indentation due to displacement creeping, which complicates the mechanical characterization of soft biomaterials.

### 4.3. Transition from Linear to Nonlinear Behavior

In general, the assumption of small strain need to be satisfied when characterizing soft biomaterials based on Hertz contact model, so it is not trivial to establish criteria for linear behavior threshold. Albeit some plausible criterions have been proposed empirically, such as a threshold of 10% indentation strain [97], it remains vague and unclear for the transition from linear to nonlinear stress–strain behavior [15]. Actually, the ranges of linearity differ from different materials and even testing methods. For example, some studies limit the strain values below 1% to guarantee the linear behavior for brain tissue via dynamic shearing testing [48,98]. As for indentation of brain tissue, this level is increased up to 10% strain by Elkin et al. [99,100], or even 45% strain adopted by MacManus et al. [31]. More recently, Budday et al. concluded different linear ranges of brain tissue in one study, in which the threshold was limited to 10% strain in compression or tension, and 20% in shear [101].

The situations are equally complicated when it comes to microscale. Leipzig and Athanasiou characterize elastic behavior of chondrocytes at ~30% strain via compression [102], and a lower level (15% strain) is suggested to be applicable in AFM indentation according to Darling et al. [103]. More strictly, this value is limited to 5% by Chen and Lu [80].

Hence, some criteria of linear behavior threshold should be referred cautiously, especially for some high levels, and be explored specifically depending on different materials and testing methods. Additionally, some correction factors or new models, extending beyond the Hertzian and linear elastic models, can be developed and utilized to minimize the above issues [80].

### 4.4. Adhesion and Point of Contact (POC)

**Figure 6:**
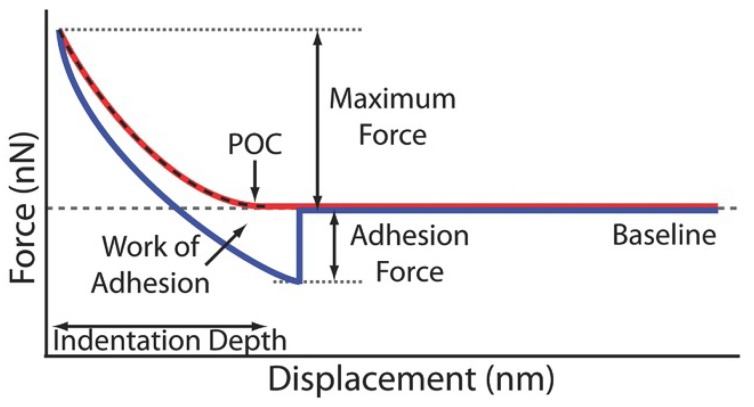
Schematic diagram of the adhesion in AFM nanoindentation, in which the red line is the approach and the blue line is the retract curve. Reproduced with permission from [86].

Adhesion, the most prevalent form of tip–sample interaction in the indentation of soft biomaterials, may omit the true point of contact erroneously, which in turn interferes the measurements of mechanical characterization (Figure 6). It is suggested that the adhesion between the tip and the sample is a significantly important parameter and needs to be taken into account for mechanical characterization, whether for a dedicated nanoindenter or AFM indentation [104,105].

When adhesion is present, some non-interactive contact models, such as the aforementioned Oliver–Pharr and Hertz contact models, may need to be modified to make the modulus values more accurate [106]. This was pioneered by JKR (Johnson–Kendall–Roberts) theory [107], which introduces an apparent Hertz load or the equivalent load. Subsequently, a seemingly contradictory theory, DMT (Derjaguin–Muller–Toporov) theory [108], was proposed, which is assumed to follow the Hertz model. Actually, The above two theories were identified in terms of sample compliance adhesive force range, in which JKR theory describes the case of relatively compliant materials with large contact size and adhesive force, and DMT theory stands the opposite with stiff materials, small contact size, and adhesive force [109]. Further, the adhesive contact mechanics was developed by Maugis–Dugdale (MD) theory, which is an intermediate case spanning the JKR and DMT limits [110,111].

Aside from the theories based on adhesive contact, some methods for non-adhesive contact have been employed for POC determination, and summed up in the literature [86,87]. These include, but are not limited to, visual inspection [112], model fitting [105], extrapolation [112,113], and Bayesian analysis [114]. Additionally, another strategy has been considered to characterize mechanical behavior without needing to determine the POC. A method was initially proposed by A-Hassan et al. to compare a known reference sample [115]. Later a protocol of data processing to linearize the Hertz and Sneddon equations for different tips has been developed and used widely [33,116]. According to Equations (8)–(10), shear modulus *G* can be determined by measuring the slope of indentation force for differen tips *P*_cylinder_, (*P*_sphere_)^2/3^ and (*P*_cone_)^1/2^ versus indentation depth *h*:

(14) $$G_{\mathrm{cylinder}}=\frac{1}{4}\mathrm{slope}\frac{1-\nu }{R}$$

(15) $$G_{\mathrm{sphere}}=\frac{3}{8}{\mathrm{slope}}^{3/2}\frac{1-\nu }{\sqrt{R}}$$

(16) $$G_{\mathrm{cone}}=\frac{\pi }{4}{\mathrm{slope}}^{2}\frac{1-\nu }{\tan\alpha }$$

In this way, the shear modulus, defined in terms of remaining constant parameters of tip geometric and Poisson’s ratio, can be calculated without POC.

### 4.5. Viscoelasticity and Poroelasticity

It is known that almost all soft biomaterials exhibit time-dependent behavior. Apart from some aforementioned typical indentation methods to characterize time-dependent behavior, dynamic testing, where a sinusoidal load rather than a trapezoidal load is applied to measure storage and loss modulus directly as a function of loading frequency, is also widely used [117,118].

Consider that many soft biomaterials exhibit time-dependent behavior under specific physiological conditions, some explicit analysis connected with physiological conditions are more likely to be central to future studies of soft biomaterials, rather than considering water solely as a solvent or an adaptive component. Compared with linear viscoelasticity, poroelasticity can clearly model the multiphase nature of soft biomaterials. Additionally, different from the empirical fitting of linear viscoelastic model, poroelastic model is mechanistic and can relate the rheological properties to structural or biological parameters, which in turn can predict the changes in rheology due to microstructural changes [32]. It also need to be mentioned that these two effects (viscoelasticity and poroelasticity) act relatively independently and can be separated uniquely with exhibiting both behaviors simultaneously [119]. This independence makes sense since one mechanism is dictated by tissue dissipation and the other by fluid flow [26].

In the case of some complicated constitutive response, such as a nonlinear viscoelastic or a poroelastic model, there may be no closed-form analytical expression of the indentation issue, and some approaches of finite element analysis (FEA) are proved to be useful [90]: inverse optimization FEA model [120], approximation of forward FEA simulations [121], and a fitted database [122]. In fact, no matter what constitutive model is used to characterize time-dependent behavior, most data analysis are performed “off-line” from the instrument software, which impedes development of commercial instruments, especially in the field of biomaterials characterization. More details about the comparisons between viscoelastic model and poroelastic model can be seen in [82].

### 4.6. Sample Hydration and Environmental Control

Another critical factor for mechanical characterization of most soft biomaterials is the hydration state of the sample. Owing to the difficulties respect to instrument setup, data acquisition and analysis in handling the hydration state of the sample, there have been to date, relatively few studies under a hydrated state. However, a number of studies have concluded lower elastic modulus values for hydrated soft biomaterials with as much as an order of magnitude than that of the dried counterpart [123,124].

There are two different basic strategies for hydrated testing of samples: “off-line” and “on-line”. Samples can be hydrated in fluid prior to testing, and just removed from the fluid while indentation within a short time period before the fluid evaporates [88,118]. This is the simplest hydration method, but may be problematic in a way considering the susceptibility of biomaterials to ambient conversion. Alternatively, samples can also be tested in a hydrated or submerged fluid environment, including partially/fully submerged in fluid [33,42], surrounded by hydrating foam layer [29], hydrated by specialized irrigation system [51], and covered by physiological saline-coated gauze [30].

Even if the samples have been hydrated, it is still unmet for environmentally-controlled nanoindentation under the truly physiological state. First is the types of fluid environments. Sometimes, water is used for sample hydration instead of physiological saline or other special solution (such as artificial cerebrospinal fluid for brain tissue) to minimize the risk of fluid damage to the instrument, especially for commercial instruments, but such substitution may lead to alteration of mechanical response [125]. Further, most studies are performed at room temperature rather than body temperature, and even current models for mechanical characterization perform poorly when fitting such experimental data. Thus, some control of experimental design and better models are needed to characterize soft biomaterials under a truly physiological state.

Consequently, considering the complexity of physiological activity, it may be better to some extent to achieve environmentally controlled nanoindentation based on “biology” instead of “mechanics”, i.e., it is more intriguing for in vivo testing, compared with in situ or in vitro testing.

## 5. Applications

### 5.1. AFM Indentation

By virtue of its high spatial and force resolutions, AFM has long been a critical technique for characterizing biological systems, from biomolecules to complexes, which, in turn, has been a driving force for the expansion of the repertoire of novel applications [126,127,128]. Today, AFM has become increasingly sophisticated, especially combined with surface morphology and topography at the micro/nano-scale [128,129,130].

#### 5.1.1. Biomolecular Level

Mechanical characterization at the biomolecular level can provide the insight into how properties and functions at more intricate biomolecular complexes (single cells and tissues, and organelles) arise from a myriad of single biomolecules. For example, intermediate filaments offer mechanical stability to cells when exposed to mechanical stress, and act as a support when the other cytoskeletal filaments cannot keep the structural integrity of the cells [131]. AFM can also be applied for the mapping of target RNA distribution using probe DNA-immobilized AFM tips [132], which is fundamentally important to understand the regulatory mechanisms underlying cellular and tissue differentiation [133].

**Figure 7:**
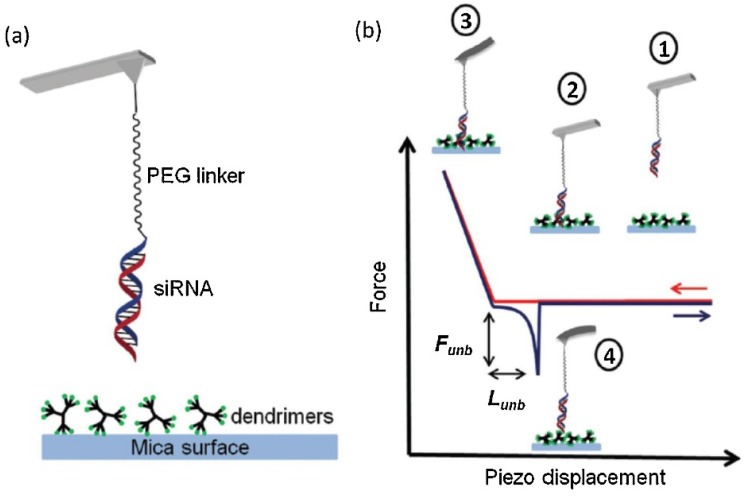
(**a**) Scheme of the tip functionalization and dendrimer adsorption on mica. (**b**) Main steps of a force curve depicting a molecular recognition (specific) event. ①. Tip far from the surface. ②. Initial tip–surface contact (approaching). ③. Tip–surface repulsive region. ④. Molecular recognition unbinding force. Reproduced with permission from [135].

In addition to the single biomolecules, interactions of molecules (Figure 7) are also able to produce significant effects on the functionality of larger-scale biomolecular complexes and tissues. DNA-protein interactions, which play important roles in numerous fundamental biological processes such as DNA replication, packing, recombination, DNA repair, RNA transport, and translation, can also be explored by AFM [134].

Further, characterization of biomolecules can also provide the potential to supplement the field of biomaterials with new resources for the design of novel materials and nanocomposites [136].

#### 5.1.2. Cellular Level

**Figure 8:**
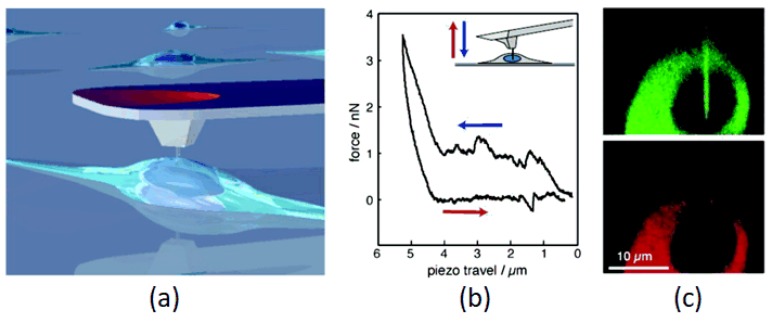
Nanoscale operation of a living cell using AFM. (**a**) Schematic representation of a nanoneedle on the AFM tip over a living cell. (**b**) Force-distance curves during approach and retraction from a melanocyte. (**c**) Cross-section images for green and red emission processed from confocal slices. Reproduced and adapted with permission from [20].

Cell mechanics, one of the most important biophysical properties, can provide new perspectives on pathologies and classic biological research questions [32]. AFM has been recognized as a powerful technique to understand cellular processes in the applications of regenerative medicine and tissue engineering [137]. Further, AFM indentation can offer various functionalities, starting from surface imaging to detection of interaction forces, delivering quantitative mechanical behavior that can describe changes characteristic in diagnosis of many pathological conditions, such as cancer [138]. Together with surface topography, AFM can also offer accurate constitutive data about surface physico-chemical properties, such as elasticity [139], friction [140], adhesion [141], and even nanoscale surgeries on live cells (Figure 8) [20].

In addition to the cell itself, the extracellular matrix (ECM) and the cell membrane involved in communications between cells and surrounding matrix also has a critical role in regulating cell properties and behavior. AFM can provide important insights on our current understanding of the mechanics of cells, ECM, and cell-ECM bidirectional interactions [142,143,144,145].

The reader interested in more details of specific cells can refer to a number of AFM indentation publications including: cardiomyocytes [146], liver cells [147], chondrocytes [148], neurons [149], fibroblasts [150], and mesenchymal stem cells [151].

### 5.2. Dedicated Instrumented Indentation

**Figure 9:**
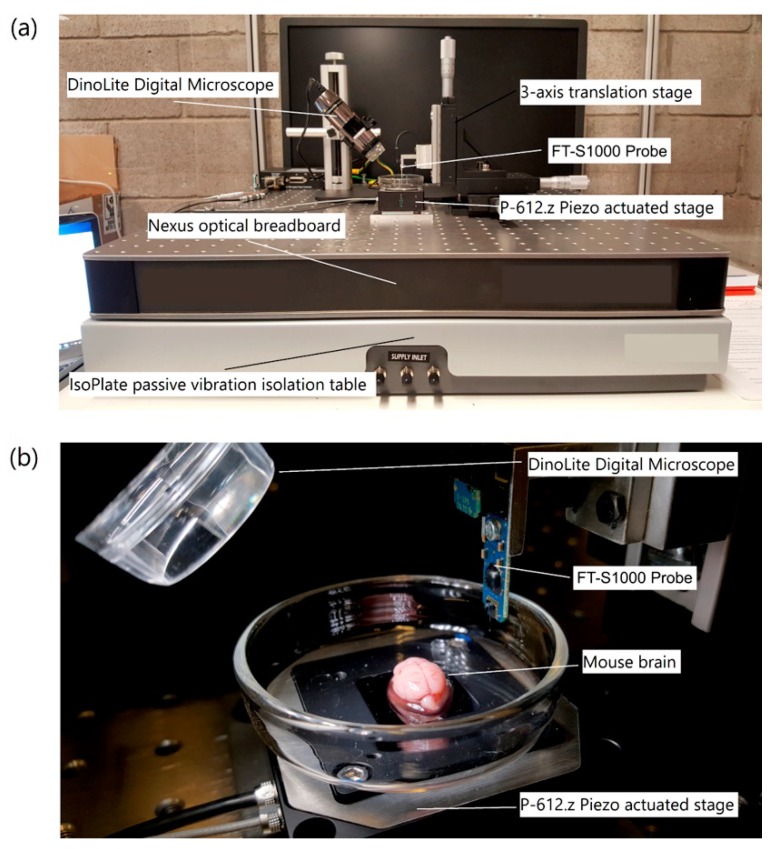
(**a**) Major components of the custom built micro-indentation experimental apparatus, and (**b**) a close-up of the force sensing probe and mouse brain specimen prior to indentation. Reproduced with permission from [153].

Owing to the indirect loading and the compliance of cantilever combined with adhesive effects of AFM indentation [90], more robust data may be provided via dedicated instrumented indentation, especially at the tissue level (Figure 9).

For example, in the field of traumatic brain injury (TBI), detailed anatomically correct geometry and accurate constitutive data of brain tissue are required to construct accurate brain models [31]. Brain models of TBI play an important role in the process of evaluating and understanding the complex physiologic, behavioral, and histopathologic changes associated with TBI, which, in turn, is necessary to establish numerical predictions and new therapeutic strategies in brain injury [152].

Indentation technique can also be utilized in the fields of tissue replacement and repair. It is shown that nanoindentation can be utilized for functional evaluation of cartilage repair tissue [30]. Owing to the in situ testing and nondestructive nature, indentation technique allows for subsequent histological or biochemical analysis of the same tissue, which offers a more complete description of repair tissue.

Other applications involved in indentation to soft tissue include, but are not limited to, poroelastic properties of articular cartilage [154], viscoelastic properties of liver tissues [155], remineralization of demineralized dentin [156], and in vivo tests of skin [157].

## 6. Challenges and Perspectives

### 6.1. Open Questions and Challenges

Nanoindentation techniques have emerged as indispensable tools for mechanical characterization, with the development of the versatility. However, apart from some inherent characteristics we have mentioned above, there are still a number of challenges which are particularly significant in soft biomaterials.

**Figure 10:**
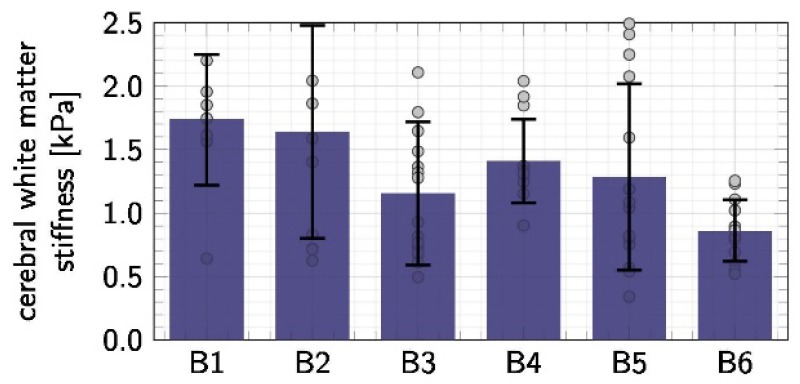
Example of deviations among individual measurements in the literature. *n* = 116 indentation tests of cerebral white matter within six brains (B1–B6). Individual measurements are indicated as dots; means and standard deviations are shown as bar graphs. Reproduced with permission from [17].

Firstly, and the most importantly, there is a paucity of consensus on the appropriate data analysis to mechanical characterization of soft biomaterials. This issue can be illustrated by twofold factors:

- Deviations among individual measurements in one research, owing to diversities in tissue architecture and structural heterogeneity (Figure 10).
- Very large disparities in the results reported in different studies, essentially linked with variations in the protocols [158].

As such, it remains difficult to characterize and model soft biomaterials accurately, even though there is a very large amount of information regarding mechanical characterization. The influence of the first factor which is attributed to inherent complexities of soft biomaterials, can be reduced to a certain extent by some repetitive experiments. In this regard, it seems to be more urgent to build a standardized routine from sample preparation to test execution, hence, properly dealing with the large disparities reported in different studies.

**Figure 11:**
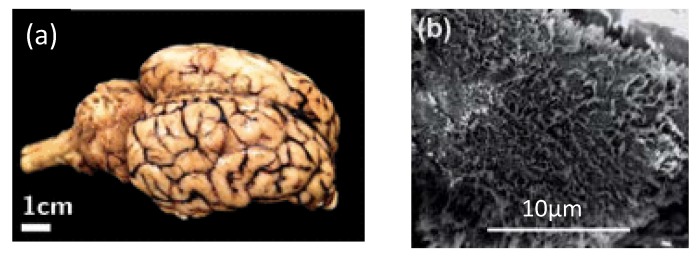
Examples of the non-ideal surface of soft biomaterials in the form of numerous asperities. (**a**) Macroscale: the surface of porcine brain tissue with sulci and gyri, reproduced and adapted with permission from [161]; and (**b**) microscale: the surface of human cervical epithelial cells with brush, reproduced and adapted with permission from [162].

Secondly, the surface of some soft biomaterial is not flat and smooth ideally that comes in the form of numerous asperities (Figure 11). It may invalidate the underlying assumption of some of analytical theories, as well as bring some difficulties to test operation and data analysis, especially for a dull indenter [72]. In this case, a relative large indentation depth may be applicable to some extent [159,160]. It need to be mentioned that although some additional processing and preparation methods (e.g., cryomicrotoming) can be adopted to reduce surface roughness, the mechanical properties of the sample may be affected by the preparation or processing mechanism. Further, it seems to be more fascinating for biomaterials to interpret the mechanical behavior thereof with original morphology, i.e., in situ and in vivo tests.

Thirdly, some assumptions of analytical relationships, such as isotropy and homogeneity, are poor descriptions and are never perfectly met in practice. The application of above analytical models may lead to certain degree of error herein. Since these aspects are directly related to the microstructural features and variations thereof, an analytical model that could simultaneously account for all involved complexities of soft biomaterials, such as nonlinear behavior, time-dependence, anisotropy, and heterogeneity, may be far-reaching, but also be daunting due to the very large workload and specialized knowledge.

### 6.2. Perspectives and Opportunities

Despite significant progress based on current research-driven user base, there is a general agreement that a more detailed understanding of mechanics involved in accurate constitutive data and anatomical distribution is required, thereby offering more opportunities for medical field such as pathology and biochemistry.

For many soft biomaterials, mechanical characterization under in vivo environment may have more important consequences in understanding the physical biology. Compared with the test ex vivo, some alterations in mechanical properties may exist due to perfusion pressure, tissue degradation, and boundary condition effects [158]. More recent study indicates that the mechanical properties change drastically within only several minutes post mortem [163]. Accordingly, in vivo testing may be a better (even the only) strategy to provide insight into mechanical behavior of some soft biomaterials.

The investigation of the testing under real service conditions and even some extreme environment conditions is another critical step for future research. In fact, most soft biomaterials operate at multiple and complicated loading conditions, and thus some measurements under ideal experiment condition may overlook many potential details. In particular, some results derived under one loading-boundary mode does not necessarily conform to the material response under another mode [161]. In addition, the biomaterials under some extreme environment conditions, such as high electric field, high magnetic fields, intense radiation, and extreme low/high temperature, may have some novel behavior [164], which, in turn, provide a better understanding for the application in some fields, such as medical research and space exploration.

Compared with most researches focused on the mechanical characterization, naoindentation can also be developed as a powerful tool in the field of mechanobiology [165]. Recent findings have directly linked development, cell differentiation, physiology, and disease to mechanical response both at the cellular and tissue levels [166]. Insights into the mechanical signal transmission from the macroscale to the nanoscale would allow us to better understand composition–structure–properties relationships in biomaterials.

Elastographic techniques (e.g., magnetic resonance and ultrasound elastography), also acting as noninvasive tools, can quantify the mechanical behavior of soft biomaterials in vivo as well [163].

Despite the infancy of these techniques, lacking a thorough verification and validation [167], it is expected that broad application prospects will arise in the fields of various pathologies if connected with nanoindentation techniques.

## 7. Conclusions

In this review, we have discussed the topic of nanoindentation of soft biological materials. Owing to some characteristics of soft biomaterials, including nonlinearity, time-dependence, anisotropy, and heterogeneity, some test protocols regarding constitutive model and data analysis are different from typical engineering materials. Although a standardized routine for nanoindentation of biomaterials remains to be built, nanoindentation techniques have emerged as indispensable tools for investigation of both biomechanics and mechanobiology. The applications of nanoindentation have contributed far more than simply characterizing mechanical behavior from bulk scale to the micro/nano-scale. There will be more opportunities for their use in other fields, such as physiology and pathologies.

## Figures and Tables

**Table 1:** Comparisons between the Oliver–Pharr method and Hertz contact model.

	Oliver–Pharr Model	Hertz Contact Model
Application	Typical engineering materials and hard biomaterials	Soft biomaterials
Tip Selection	Typically using sharp indenters (e.g., Berkovich and Vickers)	Typically using dull indenters (e.g., spherical and flat-ended)
Control Mode	Typically using load-control	Typically using displacement-control
Data Analyzed	Unloading	Loading (include holding when characterizing viscoelasticity)
Method for Time-Dependence	“Correfcted” Oliver–Pharr analysis	Boltzmann hereditary integral (Equations (11)–(13))

**Table 2:** Comparison between sphere and flat-ended tip for characterization of soft biomaterials

	Sphere Tip	Flat-Ended Tip
Advantages	Can offer the stress without high stress concentration at the contact perimeter.	Can simplify data analysis within a constant contact area during indentation. Can achieve more force value compared with sphere tip at the same depth, which is crucial to the instruments with low signal-to-noise ratios.
